# Gastric schwannoma with giant ulcer and lymphadenopathy mimicking gastric cancer: a case report

**DOI:** 10.1186/s12876-020-01186-2

**Published:** 2020-02-14

**Authors:** Caihua Tang, Qiyong Pan, Zeqing Xu, Xuan Zhou, Ying Wang

**Affiliations:** 1 Department of Nuclear Medicine, The Fifth Affiliated Hospital, Sun Yat-sen University, Zhuhai, 519000 China; 2 Department of pathology, The Fifth Affiliated Hospital, Sun Yat-sen University, Zhuhai, 519000 China

**Keywords:** Gastric schwannoma, Positron emission tomography, FDG, Ulcer

## Abstract

**Background:**

Gastric schwannomas are rare benign tumors originating from the intramuscular plexus of the stomach and account for just 2.6% of gastric mesenchymal tumors. Gastric schwannoma (GS) with a surface ulcer is very rare. Herein, we report a rare case of an ulcer-bearing GS, which in conjunction with multiple enlarged regional lymph nodes, readily mimicked gastric cancer (GC).

**Case presentation:**

A 79-year-old female presented with poor appetite and intermittent vomiting of gastric contents during the past month. Gastroscopy revealed a giant crateriform ulcer within the stomach body (at the angular notch). Its raised and indurated border was fragile and bled easily. GC was thus suspected. Contrast-enhanced computer tomography (CT) revealed a mild enhancement of the corresponding irregularly thickened gastric wall, and an annular zone of mucosal discontinuity. Enlarged regional lymph nodes were also found, making GC with metastases of lymph nodes our primary concern. ^18^F-fluorodeoxyglueose position emission tomography (^18^F-FDG PET)/CT was then performed for further staging. Obviously increased FDG uptake was shown in the gastric lesion ((maximum standardized uptake value (SUV_max_) 14.6), but no FDG uptake was observed in the enlarged regional lymph nodes. Given the strong suspicion of GC, subtotal gastrectomy was performed. GS was revealed by postoperative pathology, with no evidence of metastasis in the 13 resected lymph nodes.

**Conclusions:**

This was a rare case of GS with a giant surface ulcer and multiple enlarged regional lymph nodes. The uptake of ^18^F-FDG in the tumor was substantially higher than previously published literature reports. Under these circumstances, it is difficult to be differentiated from GC.

## Background

Schwannoma is a benign tumor originating from the nerve sheath that rarely involves the digestive tract. Gastric schwannomas arise from the intramuscular plexus of the stomach wall, accounting for just 2.6% of gastric mesenchymal tumors and only 0.2% of gastric neoplasms [1]. Herein, we report a rare case of gastric schwannoma (GS) that mimicked gastric cancer (GC). A giant surface ulcer and resultant lymphadenopathy created a false impression of malignancy.

^18^F-fluorodeoxyglueose position emission tomography (^18^F-FDG PET) has been used extensively for the differential diagnosis of benign or malignant tumors and the FDG uptake has been demonstrated to have a significant correlation with the malignant potential of various tumors [2]. ^18^F-FDG PET imaging of GS has only been reported in a few cases, all demonstrating increased FDG uptake ranging from SUV_max_ of 3.3 to 7.1 [3–7]. In our patient, the tumor showed an obviously increased FDG uptake (SUV_max_ 14.6), which was significantly higher than that of other cases reported in the literature.

## Case presentation

**Fig. 1:**
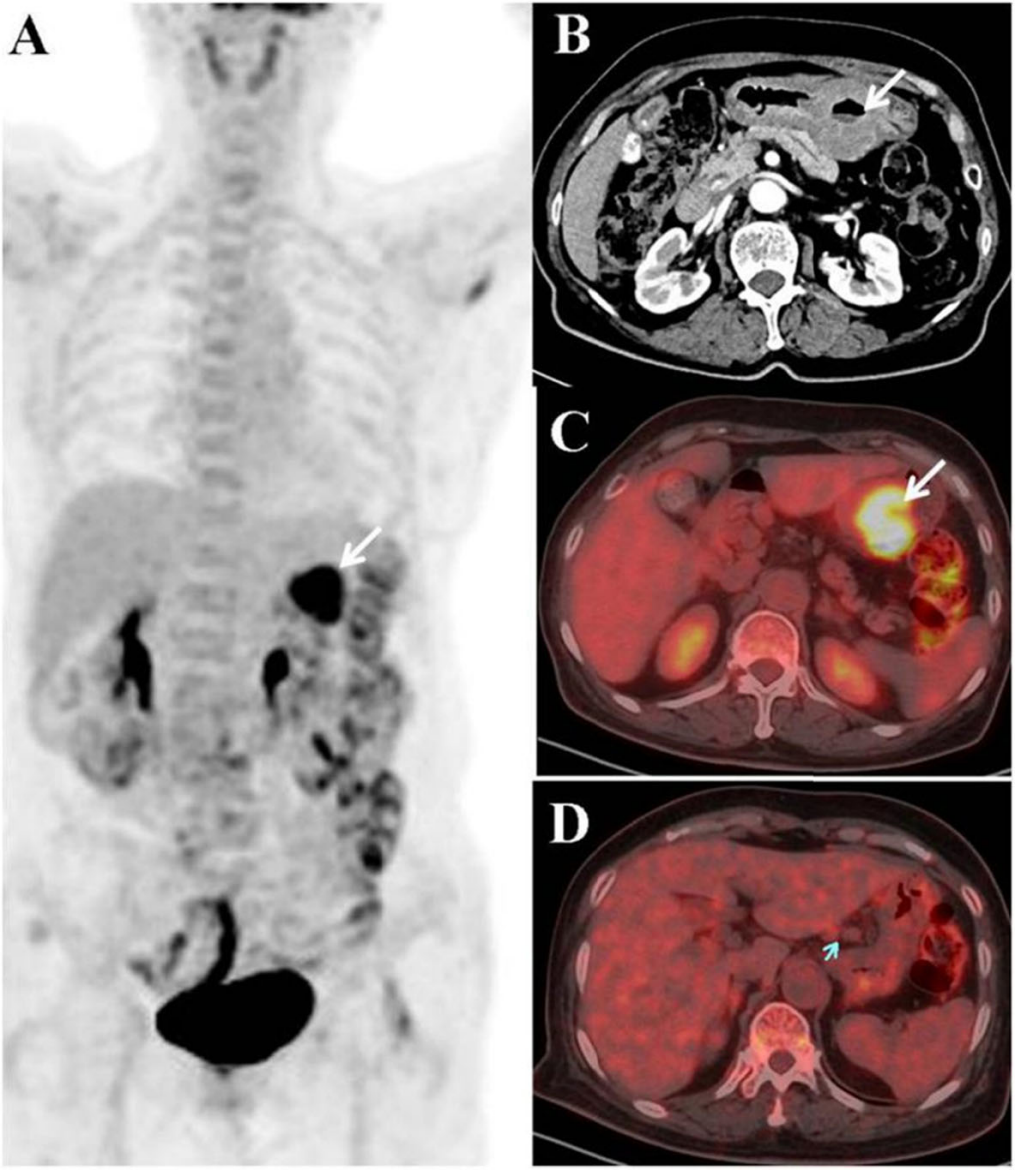
Radiographic details **a**^18^F-FDG PET shows a high accumulation of FDG in left upper abdomen (white arrow). **b** Contrast-enhanced CT view of the irregularly thickened gastric wall with slight enhancement. **c** Increased FDG uptake was observed in the gastric mass (SUV_max_ 14.6, white arrow). **d** Enlarged lymph nodes abutting the lesser curvature of the stomach, lacking FDG intensity (blue arrow)

A 79-year-old female presented with poor appetite and intermittent vomiting of gastric contents during the past month. A regimen of proton pump inhibitors offered no major symptomatic relief. Gastroscopy revealed a sizeable (4.5 cm × 6.0 cm) crateriform ulcer within the body of the stomach, at the angular notch. The raised, indurated border was fragile and bled easily. Thus, GC was suspected. Histopathology after biopsy showed inflammatory granulation tissue, exudation and necrotic material, and no malignant tumor cells were found. Contrast-enhanced computed tomography (CT) of the upper abdomen showed a mild enhancement of the gastric body, accentuating an irregularly thickened wall. An annular zone of mucosal discontinuity was also identified (Fig. 1 b), and a number of enlarged lymph nodes were noted in the vicinity (Fig. 1 d), the largest one measuring 12 mm × 9 mm. Malignancy was strongly suspected, either GC with multiple nodal metastases or lymphoma.

Routine blood, urine and stool tests were all within normal ranges, as were indices of liver and kidney function. Serum assays for various tumor markers were negative. ^18^F-FDG PET/CT was performed for further staging purposes. The images disclosed a protuberant soft tissue mass in the gastric cavity, with a heterogeneous increase in FDG uptake (maximum standardized uptake value (SUV_max_) 14.6). However, no FDG uptake was observed on the enlarged perigastric lymph nodes (Fig. 1 d). Malignancy was still our chief concern, especially GC, although the weak nodal uptake suggested a reactive process.

**Fig. 2:**
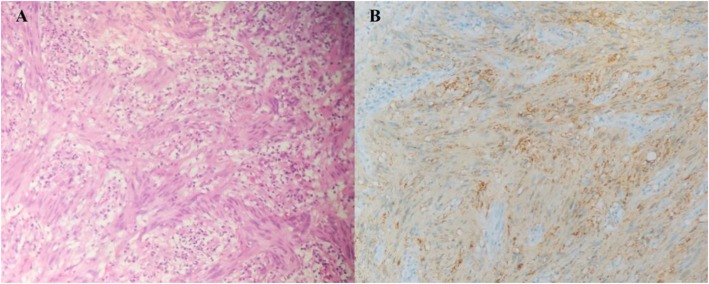
Hispathological findings: **a** Spindled tumor cells with large nuclei and vague nuclear palisading (H&E, 100 ×). **b** The spindle tumor cells shows a positive reaction for S-100 protein by immunohistochemical staining (100 ×)

Subtotal laparoscopic gastrectomy was subsequently performed. GS was revealed by postoperative pathology. Histopathology revealed a mass with necrosis on the surface. The lesion was sallow, firm, and deeply entrenched in the muscular layer, without breaching serosa. Hematoxylin and eosin (H&E) stained slides revealed abundant spindle cells. The Ki-67 protein level, reflecting cellular proliferative activity, was < 5%. Immunohistochemical examination showed that S-100 and SOX10 were positive (Fig. 2 b), whereas CK7, CK20, CK, villin, CDX-2, CD117, CD34, SMA, desmin, and c-erbb-2 were negative. No tumor metastases were seen in any of the sections of the excised lymph nodes (0/13). The patient has done well one year following surgery, showing no signs of recurrence or metastases.

## Discussion and conclusion

Schwannoma usually originates from the Schwann cell of the peripheral great nerve trunk, spinal nerve or cranial nerve, and rarely involves in the digestive tract. Gastric schwannomas are usually found in elderly women, and the most commonly referred symptom is abdominal discomfort, while upper gastrointestinal bleeding can be accompanied in patients with mucosal ulcer [8]. The tumors are usually benign, slow-growing, and have good prognosis [1]. In the present case, the patient has done well one year following surgery, showing no signs of recurrent or metastatic diseases.

GS must be differentiated from other submucosal tumors such as gastrointestinal stromal tumors (GISTs) [9], which originate from mesenchymal tissue as well. The locations, symptoms and growth patterns are frequently indistinguishable. The range of ^18^F-FDG uptake in malignant GISTs is partly shared with that of GS. Nevertheless, necrosis and cystic degeneration are more common in the former, and local and distant metastasis can also be seen in malignant GISTs, while such features are very rare in GS. Mild to moderate, uniform, progressive enhancement is instead characteristic of GS [10], and enhancement of GISTs is generally higher than that of GS in contrast-enhancement CT scans. A diagnosis of GS relies on histopathological examination. GISTs consist of spindled cells with nuclear palisading and may mimic schwannomas in particular. However, the immunohistochemical staining between these spindle cell tumors are different [11]. Positive CD34 and CD117 indicate GISTs, and positive S-100 indicates schwannomas. In the present case, the tumors revealed spindle cells, which were strongly positive for S-100 and SOX10 staining, and negative for CD117 and CD34, thus indicated the diagnosis of schwannomas.

At times, GS must be differentiated from GC, which often occurs at the lesser curvature of the stomach, creates an irregularly thickened wall, has indistinct borders, directy invades adjacent tissues, and spreads to lymph nodes [12]. Degrees of ^18^F-FDG uptake partly overlapp in these two diseases, so the distinction was mainly based on CT morphology. However, distant metastasis and adjacent invasion can be detected by PET/CT, which helps us to differentiate and diagnose. This case was preoperatively misdiagnosed as GC, mainly due to the irregular contours, extensive surface ulceration, and the remarkable intensity of ^18^F-FDG uptake. The prominent perigastric lymph nodes, with weak ^18^F-FDG uptake, may have reflected the extent of inflammation involved. Repeat deeper biopsy guided by endoscopic ultrasonography may be performd preoperatively, which was not considered because the ulcer border was fragile and bled easily. Maybe, it was the limitation of this case. We expect our case report would contribute to the recognition of the biological chracteristics of GS and to avoid inappropriate under- or overtreatment of the patients in the future.

^18^F-FDG PET/CT has been used extensively for the detection, staging, therapeutic monitoring, and follow-up of a variety of malignant tumors [2]. The FDG uptake has been demonstrated to have a significant correlation with the malignant potential of GISTs [9]. To the best of our knowledge, only a few cases of gastric schwannoma have been reported with ^18^F-FDG PET/CT. Komatsu et al. [5] were the first to report a patient with GS who exhibited increased FDG uptake. Yap et al. [6] described a patient with histologically confirmed GS and non-Hodgkin’s lymphoma.^18^F-FDG PET/CT was performed before and after treatment for lymphoma, showing an increased uptake within the gastric lesion (SUV_max_ 4.9). Takeda et al. [7] reported a patient with GS at the greater curvature of the body of the stomach. The lesion was rounded, showing inward growth, smooth edges, uniform density, and progressive enhancement in CT images. Increasing uniformity of ^18^F-FDG uptake (SUV_max_ 6.28) was observed by PET/CT. In our patient, FDG uptake (SUV_max_ 14.6) by the tumor was significantly higher than that of other cases reported in the literature. This value also far exceeds the SUV_max_ cutpoint of 6.1 established by Benz et al. [13] for separating malignant from benign peripheral nerve sheath tumors (PNSTs). However, the benign histopathology features of the tumor in our patient refutes their stated threshold. Therefore, these data suggest that ^18^F-FDG PET/CT has a limited role in making preoperative differential diagnosis between benign schwannoma and malignancy.

Schwannomas originating in the stomach are seldom associated with ulceration. Oh et al. [14] identified a GS within the submucosa of the lesser curvature of the stomach by gastroscopy, which with a central ulcer was misdiagnosed as a gastric malignant stromal tumor. Increased uptake of ^18^F-FDG (SUV_max_ 7.1) of the tumor was found by PET/CT. In the present case, a giant (4.5 cm × 6.0 cm) crateriform ulcer of the stomach at the angular notch was shown by gastroscopy, and tumor uptake of FDG was inordinately high, implying a high risk GC. However, the weak uptake shown by enlarged regional lymph nodes attested to be a reactive process. Although GS is a benign tumor, the FDG uptake of GS was significantly increased, which may be explained by the observed tumor influx of a number of inflammatory cells and the active expression of glucose transporter 3 and 1 in the tumor cells [15]. Further studies are needed to confirm the ^18^F-FDG uptake mechanism of GS.

In conclusion, this is a rare case of GS with giant surface ulceration and enlarged regional lymph nodes. The uptake of ^18^F-FDG by the tumor was significantly higher than levels previously reported in the literature. Ordinarily, GS must be differentiated from GIST, with GC becoming an added concern if surface ulceration and nodal enlargement are present.

## Data Availability

To protect the patient’s privacy, images and data of the current study are not publicly available.

## Abbreviations

CT: Computer tomography
FDG: Fluorodeoxyglucose
GC: Gastric cancer
GISTs: Gastrointestinal stromal tumors
GS: Gastric schwannoma
MIP: Maximum intensity projection
PET: Positron emission tomography
PNSTs: Peripheral nerve sheath tumors
SUV_max_: Maximum standardized uptake value
